# Glucagon-Like Peptide-1 (GLP-1) Analog Liraglutide Inhibits Endothelial Cell Inflammation through a Calcium and AMPK Dependent Mechanism

**DOI:** 10.1371/journal.pone.0097554

**Published:** 2014-05-16

**Authors:** Nadia M. Krasner, Yasuo Ido, Neil B. Ruderman, Jose M. Cacicedo

**Affiliations:** 1 Diabetes and Metabolism Research Unit, Department of Medicine and Section of Endocrinology, Boston University School of Medicine, Boston, Massachusetts, United States of America; 2 Program of Nutrition and Metabolism, Department of Medicine, Boston University School of Medicine, Boston, Massachusetts, United States of America; University of Pittsburgh School of Medicine, United States of America

## Abstract

Liraglutide is a glucagon-like peptide-1 (GLP-1) mimetic used for the treatment of Type 2 diabetes. Similar to the actions of endogenous GLP-1, liraglutide potentiates the post-prandial release of insulin, inhibits glucagon release and increases satiety. Recent epidemiological studies and clinical trials have suggested that treatment with GLP-1 mimetics may also diminish the risk of cardiovascular disease in diabetic patients. The mechanism responsible for this effect has yet to be determined; however, one possibility is that they might do so by a direct effect on vascular endothelium. Since low grade inflammation of the endothelium is an early event in the pathogenesis of atherosclerotic cardiovascular disease (ASCVD), we determined the effects of liraglutide on inflammation in cultured human aortic endothelial cells (HAECs). Liraglutide reduced the inflammatory responses to TNFα and LPS stimulation, as evidenced by both reduced protein expression of the adhesion molecules VCAM-1 and E-Selectin, and THP-1 monocyte adhesion. This was found to result from increased cell Ca^2+^ and several molecules sensitive to Ca^2+^ with known anti inflammatory actions in endothelial cells, including CaMKKβ, CaMKI, AMPK, eNOS and CREB. Treatment of the cells with STO-609, a CaMKK inhibitor, diminished both the activation of AMPK, CaMKI and the inhibition of TNFα and LPS-induced monocyte adhesion by liraglutide. Likewise, expression of an shRNA against AMPK nullified the anti-inflammatory effects of liraglutide. The results indicate that liraglutide exerts a strong anti-inflammatory effect on HAECs. They also demonstrate that this is due to its ability to increase intracellular Ca^2+^ and activate CAMKKβ, which in turn activates AMPK.

## Introduction

GLP-1 is a peptide hormone secreted by the L cells of the large intestine in response to nutrient ingestion. It potentiates insulin secretion, blocks glucagon release and increases satiety [1], [2], but cannot be used directly as a therapy for Type 2 diabetes due to its short (2 minute) half life. On the other hand, pharmacological mimetics have been developed with improved stability. One of these is liraglutide, in which the substitution of an Arg for Lys 34, and the addition of a C16 fatty acid at Lys 26 (using a γ-glutamic acid spacer) increases its half-life to 12 hours [3]. As does endogenous GLP-1, liraglutide potentiates insulin secretion [4], improves HbA1c [5], and reduces post-prandial glucose levels [6] in patients with Type 2 diabetes. Its effects, however, are not limited to beta cells. Liraglutide also stimulates the satiety response [7], inhibits gastric emptying [7], [8], reduces intrahepatic lipid levels [9], induces weight loss [5], and improves several markers of vascular function [10]. Interestingly, several large-scale retrospective trials have shown that GLP-1 mimetic therapies improve biomarkers of cardiovascular disease (CVD) and diminish the incidence of CVD events [11], [12], [13], [14]. Likewise, two smaller prospective studies have suggested that GLP-1 mimetics diminish endothelial dysfunction [15], [16]. Although some of these improvements could be due to enhanced insulin secretion, and secondary changes in glucose and lipid metabolism, they could also reflect more direct effects of GLP-1 mimetics on the endothelium, such as we investigate here.

It is widely held that atherosclerotic cardiovascular disease (ASCVD) can begin with endothelial dysfunction caused by pro-inflammatory stimuli in the plasma. Patients with diabetes often have elevated plasma levels of TNFα and LPS [17], [18] both of which are associated with an increased risk of ASCVD [19]. TNFα and LPS increase the expression of adhesion molecules (VCAM-1, E-Selectin) on endothelial cells [20], [21] which in turn increase the adhesion of monocytes, the first step in the diapedesis of the latter into the intimal space where atherogenesis occurs [22]. Thus curtailing the ability of TNFα and LPS to induce the expression of VCAM and E-selectin would hypothetically decrease monocyte adhesion, and presumably atherogenesis.

Liraglutide signals through the GLP-1 receptor (GLP-1R), which is expressed in many tissues including, pancreatic islets [23] and endothelium [24]. The cellular signaling mechanisms stimulated by GLP-1R agonists in pancreatic β-cells are known [25], but whether GLP-1R agonists directly induce signaling in endothelial cells that could be cardioprotective is not clear. We demonstrate here that liraglutide exerts an anti-inflammatory effect on primary human aortic endothelial cells (HAECs) by causing a sequential increase in intracellular calcium, CaMKKβ activity, and activation of AMPK.

## Methods

### Cell Culture

Human Aortic Endothelial Cells (HAECs) were purchased from Lonza and used between passages 5–7 (Lonza, Hopkinton, MA). Cells were cultured on BD primaria plates (BD, Franklin Lakes, NJ) in EGM-2 BulletKit media (Lonza, Hopkinton, MA) at 37°C in a 5.5% CO_2_ humidified incubator. Once cells became 85–95% confluent, 3–4 days post-plating, they were serum starved overnight prior to various treatment. Trypsin was used to loosen cells for passaging as per Lonza’s instructions.

### Liraglutide

American peptide (Sunnyvale, CA) cat #46-1-48A solubilized as directed.

### Calcium Assay

HAECs were cultured in EGM-2 on MatTek glass bottom culture dishes (P35G-0-14-C) and allowed to grow for three days post plating. Prior to the addition of treatment, HAECs were serum starved overnight (1/8^th^ concentration of serum of EGM-2), then treated with 2 uM Fura-2 and 1/1000 dilution of pluronic acid for 30 minutes and washed with serum starve media for an additional 30 minutes. Fura-2 is an indicator of intracellular calcium levels, when bound to calcium it excites at 340 nM and when free at 380 nM, therefore the ratio of the two indicates internal calcium levels when multiplied by the 225 nM Kd. Pluronic acid increases permeability of the cell membrane to Fura-2 to assist its uptake. Individual dishes were placed in the ionoptix microscope and focused to show individual cells for tracking selection. Ion Optix software was used to track individual cells in designated zones. Background readings were taken for between 3–5 minutes to ensure a steady state, then the treatment, 100 nM liraglutide, was added. Calcium levels continued to be tracked every 4 seconds over 30 minutes. 1 uM Ionomycin, an ionophore which releases intracellular calcium was added to indicate the maximum intracellular calcium release. It should be noted that since HAECs are a primary cell line they differ both in size and in permeability to Fura-2. As a result, baseline differences between the individual cells is not relevant, rather changes in baseline intracellular calcium after to stimulation is the relevant measurement.

### Western Blot

To determine both the cascade(s) involved in GLP-1 receptor stimulated signaling, and anti-inflammatory effects, protein quantity and phosphorylation were measured by western blot. To evaluate the cascade involved in liraglutide signaling, HAECs were treated with 100 nM Liraglutide over a time course spanning 25 minutes. Time points used were: 0, 1, 5, 10, 15, and 25 minutes after treatment addition. To evaluate the anti-inflammatory effects of Liraglutide cells were incubated with 100 nM liraglutide for 1 hour prior to the addition of inflammatory stimulus- 10 ng/mL TNFα or 2 µg/mL LPS for an additional 3 hours. These experiments were independently repeated 6 times. After the treatment, plates were placed on ice, washed with cold PBS (3x) and 80–100 uL of cell signaling lysis buffer was added to each well (1% Triton, 20 mM Tris-HCl, 150 mM NaCl, 1 mM Na2 EDTA, 1 mM EGTA, 25 mM sodium pyrophosphate, 1 mM β-glycerophosphate, 1 mM Na_3_VO_4_, 1 µg/ml leupeptin). Total protein was measure by Pierce BCA (Thermo Scientific). Between 10–18 µg of protein was loaded per sample into a NuPage 4–12% Bis-Tris gel (Life technologies NP0336BOX). Gels were run in NuPage Mops SDS running buffer (NP0001) for approximately 1.5 hrs at 150 volts. Proteins were transferred to Millipore immobilon-P membrane (IPVH00010) in NuPage transfer buffer (NP0006-1) at 25 V for 1.5 hrs and then blocked in 5% non-fat dry milk or blotto (5% fish gelatin, 3% BSA) for at least one hour at room temperature. Membranes were then incubated overnight with one of the following primary antibodies in 1/1,000 dilution: CaMKKβ from BD Bioscience (San Jose, CA), pCaMK1 (Thr177), GAPDH, VCAM-1, E-Selectin from Santa Cruz Biotechnology (Santa Cruz, CA), pAMPK (Thr172), pACC (Ser79), peNOS (Ser1177), pCREB (Ser133) from Cell Signaling Technology (Danvers, MA), tAMPK from Epitomics (Burlingame, CA), β- Actin from Sigma (St. Louis, MO), and tACC from Millipore (Temecula, CA). After washing the membrane (3x) in Tris- buffered saline (63 mM Tris–HCl, 7.3 mM NaCl) containing 0.1% Tween 20 (TBST), membrane was incubated for 1 hour with 1/10,000 diluted in TBST secondary antibody either anti-rabbit IgG horseradish peroxidase linked whole antibody from donkey (GE healthcare NA934V), or anti-mouse from sheep (GE healthcare NXA931). The membranes were then washed three times in TBST and antigen detection was performed using Thermo SuperSignal ECL, either West Pico (34080) or West Femto (34095) was used to visualize proteins. Band intensity was measure by Scion Image software and normalized to either GAPDH, or total protein for phosphorylation events.

### Monocyte Adhesion Assay

Human aortic endothelial cells were grown in primaria (BD falcon) 24 well plates in EGM-2(Lonza) with 5 mM glucose media. THP-1 monocytes were grown in 10 mM glucose RPMI-1640+10% FBS, 1% penstrep and 1% glutamine in T-75 flasks. Prior to addition of the monocytes to the HAECs, they were spun down at room temperature, 1000 rpm for 5 minutes, and resuspended in PBS where they were dyed with 1 uM CSFC (Mol. Probes/Invitrogen cat# C1157/in sterile DMSO Sigma cat#D2650) for 30 min. They were then spun down again and resuspended in serum free RPMI-1640 for addition to the treated HAECs. 100 uL of monocyte containing media was added to every well of the 24 well plate except that for the dilution curve for quantification of monocyte number. Monocytes were allowed to adhere for 30 min and then were washed away 3 times with room temperature PBS. 100 uL of EBM serum free media and 100 uL of 2% triton-X lysis buffer were added to each well to lyse the monocytes. The samples were read at excitation of 485 nm and emission of 520 nm on an Infinite M1000 from TECAN, which is a high-end multimode Microplate Reader with premium Quad4 monochromators capable of monitoring UV, fluorescence and visible absorbencies.

A standard curve and cell count was used to determine how many monocytes were present according to fluorescence level. The wells were then normalized to protein content as a marker of cell confluence/number. To visualize adhered monocytes, images were taken under phase contrast white light to visualize endothelial cells and excitation of 485 nM and emission of 515 nm for CFSC dye and then layered to show where the monocytes are adhered and their density.

### Short Hairpin RNA Expressing Lentivirus

The sequence TGAATTAAATCCACAGAAA was chosen as a target sequence for shRNA-mediated RNAi of human AMPKα1. The pSilencer 2.0 vector (Ambion; Austin, TX) was used as the template for the human U6 promoter that was cloned using the following PCR primers: forward primer (5-GAATTC-CCCAGTGGAAAGACGC-3) and reverse primer (5-GGTGTTTCGTCCTTTCCACAAGATATATAAAGGG-3). An shRNA expression cassette was created by tandem polymerase reaction of the U6 promoter template with one forward and two reverse primers as follows: forward, 5-CACCGCGCGC-CAAGGTCGGGCA-3, and reverse 1, 5-CTACACAAACT-CCACCTGTTCAGCAATACGGTGTTTCGTCC-3 and reverse 2 5-CCAAAAAAGTATTGCTGAACAGATG-GAACTACACAAACTC-3, which contains two GU pairing mutations in the sense strand. The resulting PCR product was inserted into pCR8-GW TOPO (Invitrogen) and then transferred by LR reaction to pDSL_hpUGIP shRNA lentivirus expression plasmid (ATCC). Three µg of the lentivirus plasmid was calcium phosphate-transfected along with 4.5 µg of pLp1(gag-pol), 1 µg of pLp2 (reverse), and 2 µg of pLP/VSVG (VSVG) helper vectors into 293 T cells grown in a six-well dish. Forty hours post- transfection, 18 mL of supernatant containing lentivirus was collected. The lentivirus was then purified using a kit (lentivirus production kit, ATCGbio.com, Vancouver, Canada). The HAECs were grown in 6 or 24-well plates as previous described and were infected by incubating overnight with virus (sh-scramble or shAMPK). The cells were visualized daily for GFP fluorescence (as the sh-lentiviruses co-express GFP) and then treated and harvested 3–4 days later. The virus infection efficiency was determined to by ∼87% in our cells and caused an 83% drop in total AMPK protein levels in the sh-AMPK but not the sh-scramble infected cells.

### cAMP EIA

After treatment, cells were harvested with 0.1 N HCl. Protein levels were measured by BCA to ensure at least 1 µg/µl and enzyme immunoassay performed as per manufacturer’s instruction to quantify cAMP levels with Cayman cAMP EIA kit (cat # 581001). Quantities were then normalized to sample protein level as determined by BCA.

### Statistics

Data are expressed as means +/− standard deviation. ANOVA with Tukey post tests and two tailed t test were used to analyze differences between groups of data as appropriate. p<0.05 was considered statistically significant.

## Results

To increase the likelihood that the effects observed in these studies would be physiologically relevant primary human aortic endothelial cells (HAECs) were used. Additionally, the concentration of liraglutide utilized was 100 nM, which is within the therapeutic range achieved in humans injected with 1.8 µg/day of the widely used brand of liraglutide, Victoza [26].

### Liraglutide Reduces Protein Expression of VCAM-1 and E-selectin in Response to TNFα and LPS Stimulation

**Figure 1:**
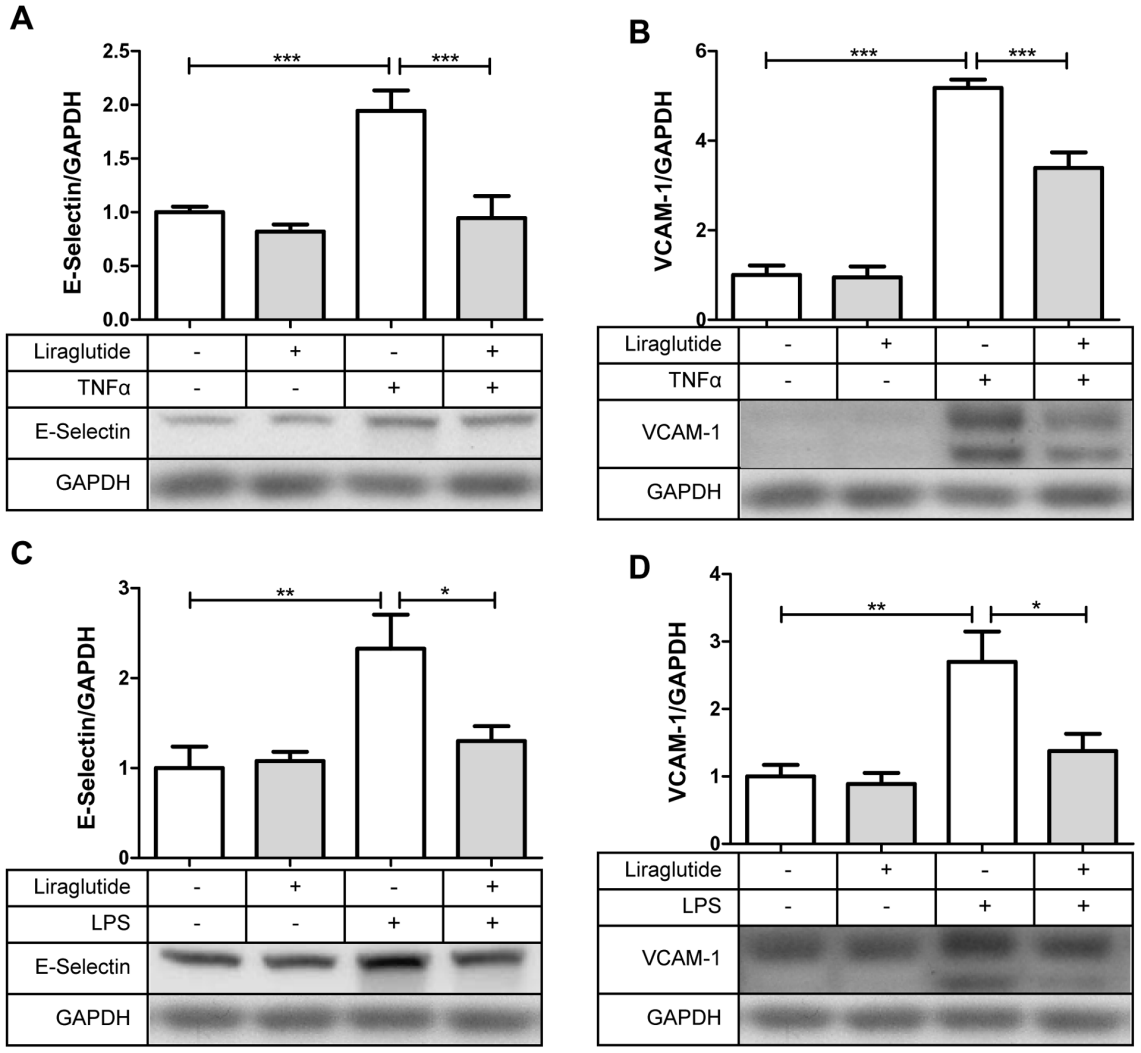
Liraglutide (100 nM) prevents the inflammatory effects (increases in cellular adhesion molecules) induced by TNFα (10 ng/mL) and LPS (2 µg/mL) in HAECs. (**A, B**) HAECs were pre-incubated for 1 hr with liraglutide and then co-incubated for 3 hrs with TNFα (**A, B**) or LPS (**C, D**) to induce VCAM-1 and E-Selectin protein expression, (n = 6). Anova with Tukey post tests, *p<0.05, **p<0.01, ***p<0.001.

Patients with diabetes have increased plasma levels of TNFα and LPS [17], [18] both of which have been linked to increased cardiovascular disease risk [19] and the induction of inflammatory responses in endothelial cells. The latter involves the expression of cellular adhesion molecules (CAMs) on the endothelial cell surface, which allows for firm adhesion of monocytes. To assess how liraglutide affects these events, HAECs were pre-incubated for 1 hr with 100 nM liraglutide, and then co-incubated with either 10 ng/mL TNFα or 2 µg/mL LPS for 3 hrs to generate an inflammatory response. As shown in Fig. 1, Liraglutide attenuated both TNFα and LPS stimulated expression of vascular cell adhesion molecule-1 (VCAM-1) and E-Selectin.

### Liraglutide Inhibits TNFα and LPS Induced Monocyte Adhesion

**Figure 2:**
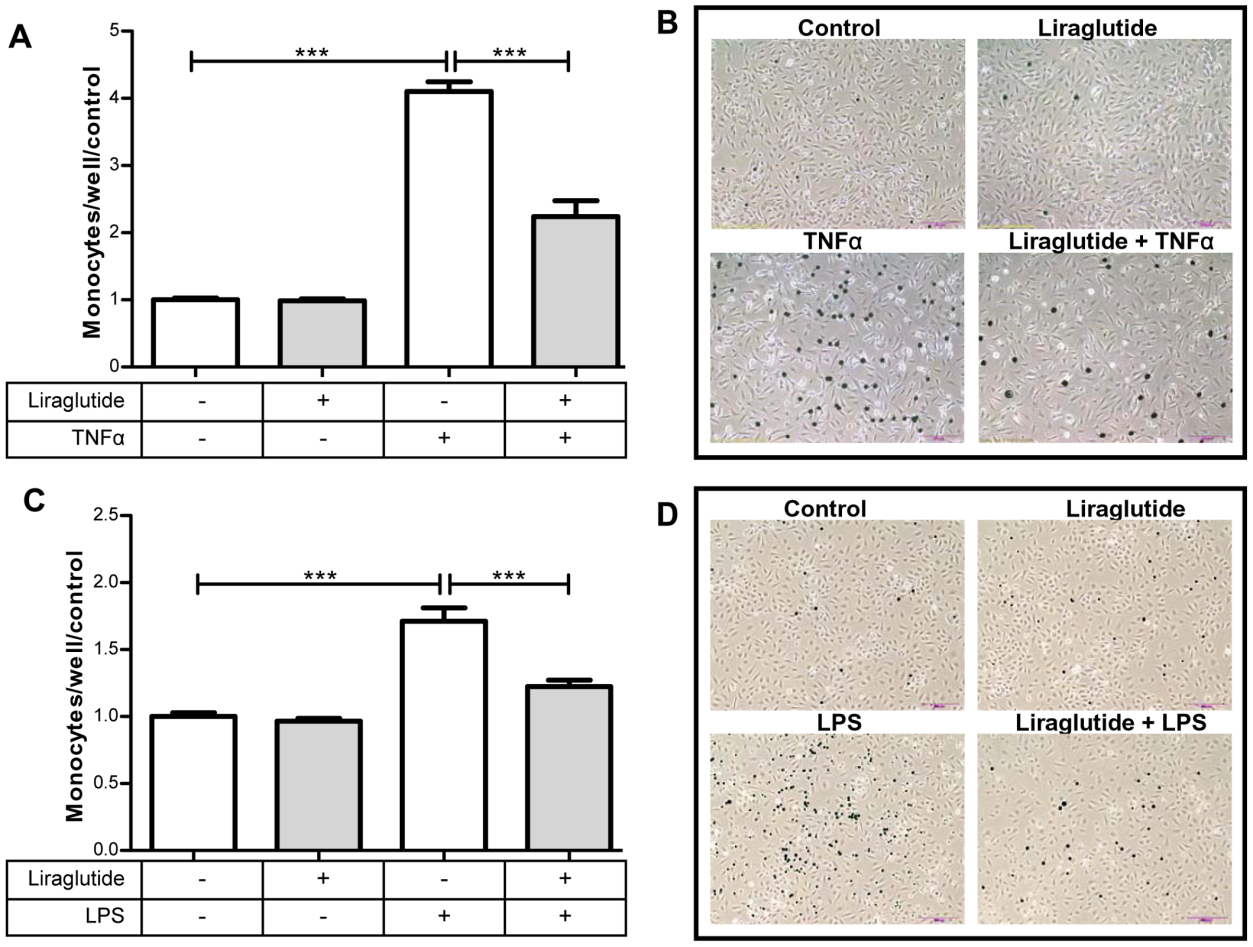
Liraglutide (100 µM) prevents TNFα and LPS induced monocyte adhesion. HAECs were pre-incubated as described in Fig.1 with liraglutide for 1 hour and, then co-incubated with either 10 ng/mL TNFα or 2 µg/mL LPS for 3 hrs. Fluorescent labeled THP-1 monocytes were then added and the incubation carried out for an additional 30 minutes. Liraglutide prevents (**A**) TNFα (n = 6) and (**C**) LPS (n = 8) stimulated monocyte adhesion. **B,D:** Representative Photomicrographs of HAECs treated as in (A and C) with adhered monocytes (visible as black dots) (10× magnification). *p<0.05, **p<0.01, ***p<0.001.

Monocytes are the largest of the cells classified as leukocytes and upon stimulation, they migrate to the vasculature, where they adhere, diapedese, and differentiate into mature macrophages [27]. Such macrophages are highly phagocytic, consume lipids and turn into inflammatory foam cells, a key event in the formation of atherosclerotic lesions [28]. THP-1 cells are a human monocytic cell line frequently used for monocyte to endothelial cell adhesion assays [29]. As shown in Figs. 2A & 2C, liraglutide (100 nM) attenuates TNFα (10 ng/mL) and LPS (2 µg/mL) stimulated THP-1 monocyte adhesion (see materials/methods). Representative pictures show fluorescent green (pseudo-colored to black) labeled monocytes adhering to treated HAECs (Figure 2B, 2D). Thus, liraglutide decreases both the expression of adhesion molecules (Figure 1) and the adhesion of monocytes to endothelial cells (Figure 2).

### Effects of Liraglutide on Intracellular Calcium and cAMP

**Figure 3:**
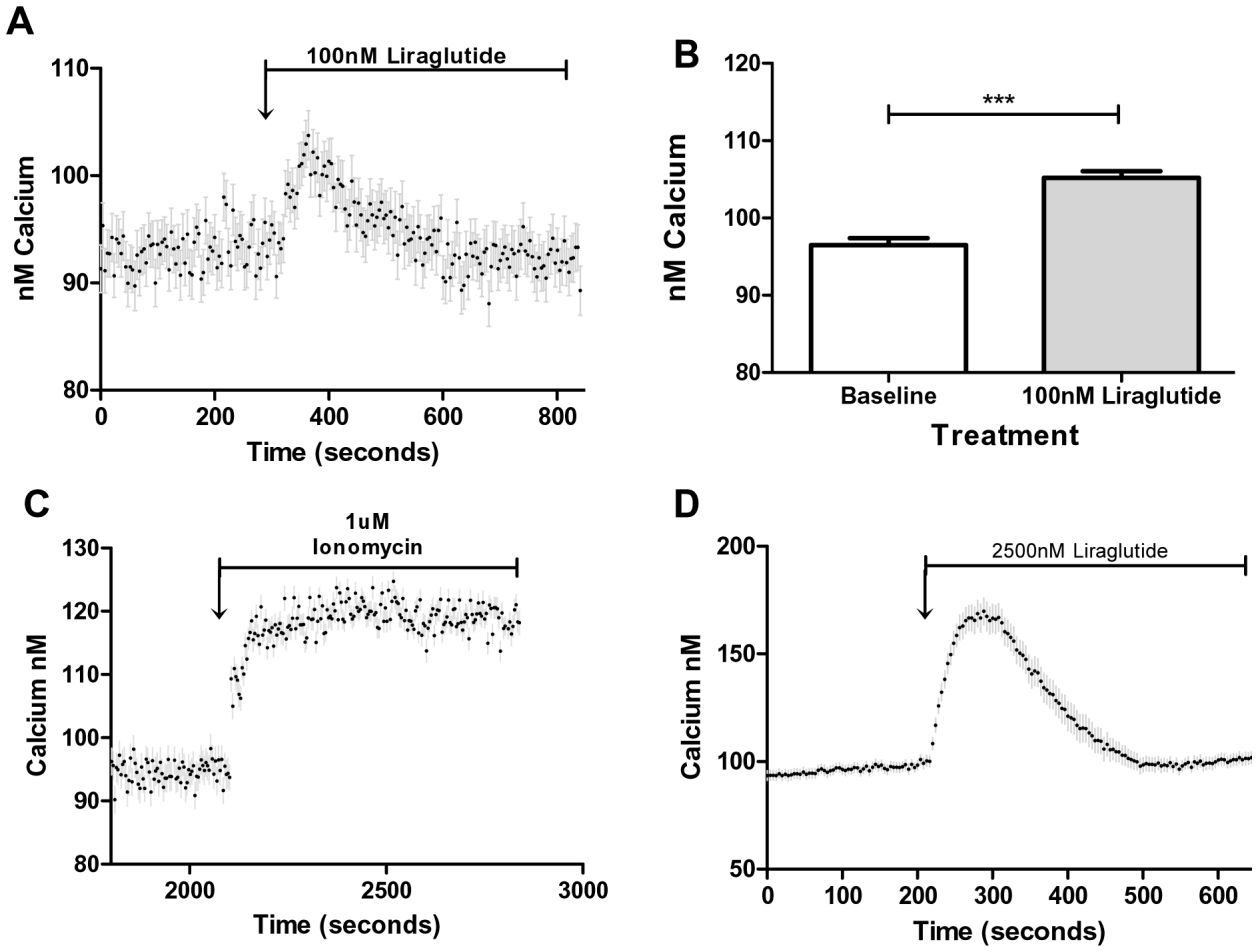
Liraglutide (100 nM) significantly increases intracellular calcium. Prior to the addition of liraglutide, HAECs were incubated with Fura-2, an indicator of intracellular calcium (See methods for details), n = 21 for all results shown. **A**: HAECs were exposed to 100 nM liraglutide where indicated by the arrow and tracked for 800 seconds. **B**: Bar graph shows intracellular calcium levels prior to 100 nM liraglutide treatment, (baseline is an average of 6 readings 236–256 seconds) and at peak intracellular calcium subsequently achieved (628–648 seconds) after its addition. **C**: Cells were tracked for 2000 seconds when 1 uM ionomycin was added to release all intracellular calcium from stores in the endoplasmic reticulum. **D**: Incubation with a supra-physiological level of liraglutide (2500 nM) caused a greater increase in cell calcium, indicating a possible dose effect for this therapy.

In pancreatic β-cells, GLP-1 signaling has been linked to its abilities to increase both cAMP and intracellular calcium [30], [31]. Calcium and cAMP are also involved in endothelial cell signaling [32], [33], [34]. Here we show that treatment with (100 nM) liraglutide significantly increases intracellular calcium levels (Figure 3), and that 2500 nM liraglutide (a supra-pharmacological concentration) causes an even greater increase in cell calcium (Figure 3D). Likewise, incubation with 100 nM liraglutide caused a small, but statistically significant, increase in intracellular cAMP levels (Figure S1), and a significantly greater increase when the cells are incubated with a supra-pharmacological concentration of liraglutide (1000 nM). Thus, liraglutide has concentration dependent effects on cell Ca^2+^ and to a somewhat lesser extent, cAMP.

### Liraglutide Activates CaMKKβ Leading to Phosphorylation of AMPK (Thr172), CaMKI (Thr177), and other Downstream Targets

To link the influx in calcium to the anti-inflammatory effects observed with liraglutide treatment, we examined other factors activated by increases in intracellular calcium that have been shown to also confer anti-inflammatory effects in endothelial cells. One of these is AMPK [35], an evolutionarily conserved fuel and stress-sensing enzyme that can be activated by calmodulin-dependent protein kinase kinase-β (CAMKKβ) [36], which itself is activated by increases in intracellular calcium, like those resulting from liraglutide treatment (Figure 3).

**Figure 4:**
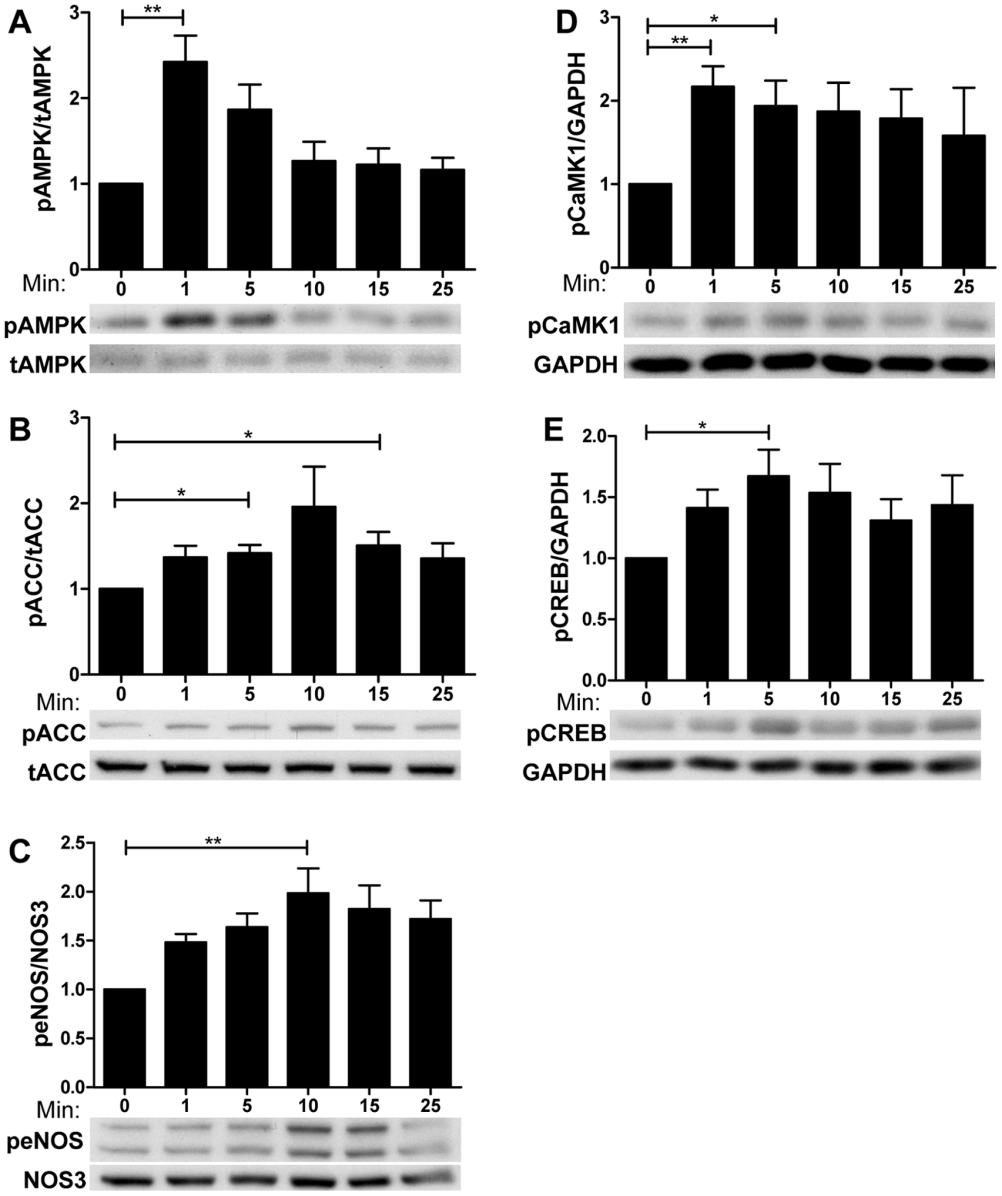
Western blot analyses of calcium sensitive and anti-inflammatory enzymes, 1–25 minutes following stimulation by 100 nM liraglutide. These graphs show the effects of liraglutide on the phosphorylation of pCaMK1 (Thr177), pAMPK (Thr172), PACC (S79) peNOS (Ser1177) and pCREB (Ser133) (n = 6) p<0.05, **p<0.01, ***p<0.001.

Time course studies revealed that liraglutide stimulates the phosphorylation of AMPK resulting in the phosphorylation of its downstream targets pACC(ser79) and eNOS (ser1177) (Figure 4A, 4B, 4C). This suggests that calcium activated CaMKKβ could be acting as an AMPK kinase. In addition to AMPK, activated CaMKKβ phosphorylates and activates CaMK1 (Thr177) (Figure 4D). This is a key observation as in addition to PKA, CREB (ser133) can be phosphorylated by CaMK1 [37], [38], as seen in Figure 4D and E. Although such serine phosphorylation of CREB could also result from the small observed increase in cAMP, inhibition of CaMKKβ, and thereby CaMK1, with the CaMKKβ inhibitor STO-609 (further discussed below) also inhibited phosphorylation of CREB (Figure S2), indicating that it is dependent on CaMKKβ activity. Moreover, although the time to peak activation seen in Figure 4 was consistent across different primary cell donors and cell passages, it occasionally ranged between 1–20 minutes for CaMK1 and AMPK with peaks for ACC, eNOS, and CREB phosphorylation occurring later.

### STO-609 Inhibits CaMKKβ Activity, Blocking Phosphorylation of AMPK and CaMKI and Inhibiting their Anti-inflammatory Effects on Monocyte Adhesion

**Figure 5:**
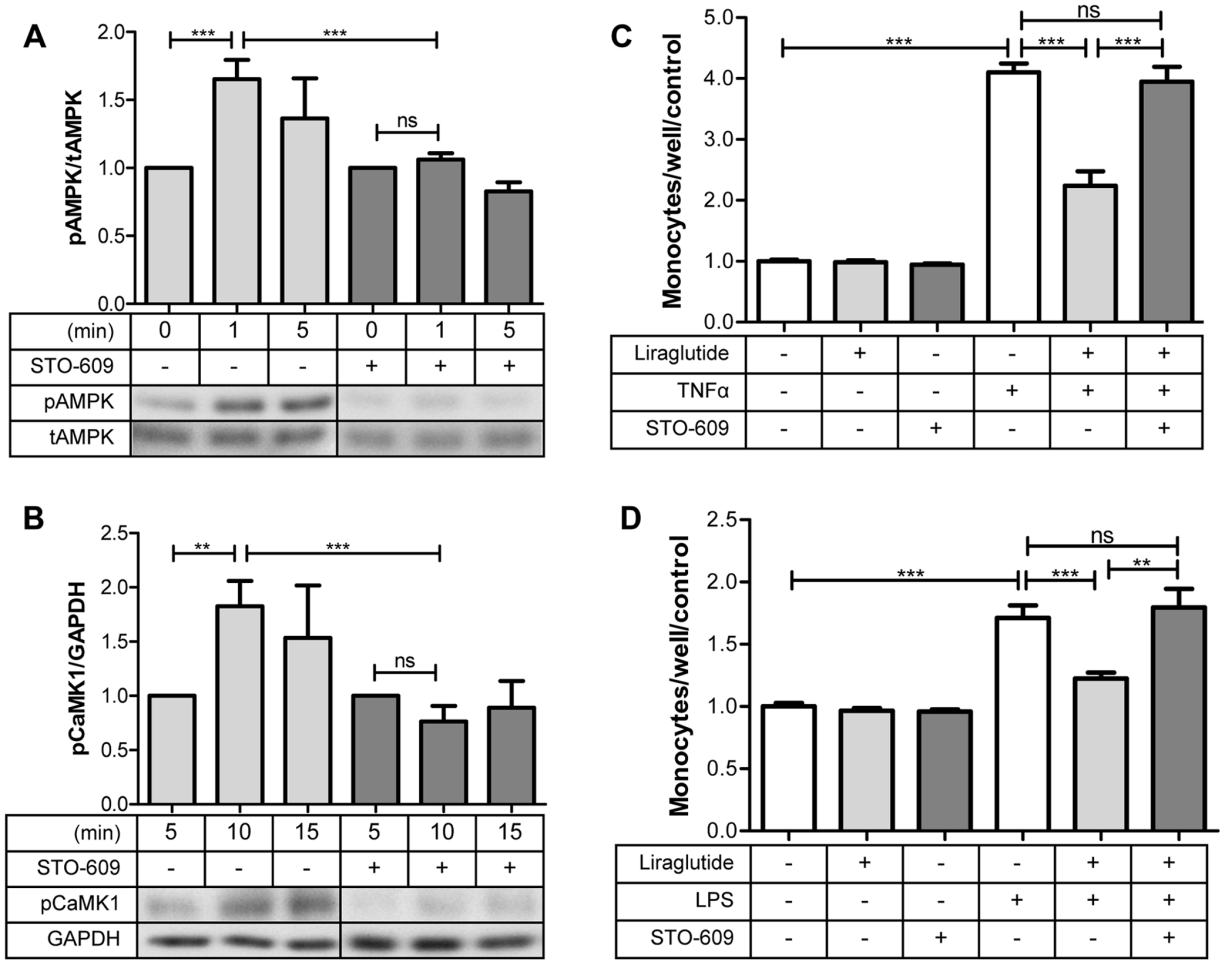
STO-609 inhibits liraglutide signaling and its effects on monocyte adhesion. Cells were incubated with STO-609 for 30 minutes prior to addition of liraglutide: **A:** pAMPK (Thr172), (n = 4**)** **B:** pCamK1 (Thr177), (n = 4) **C, D:** TNFα and LPS stimulated monocyte adhesion (n = 8). Liraglutide: 100 nM, STO-609∶0.5 µg/mL, TNFα: 10 ng/mL, LPS: 2 µg/mL. *p<0.05, **p<0.01, ***p<0.001.

To confirm that phophorylation of AMPK and CaMK1 and their downstream targets are indeed downstream of CaMKKβ activation we preincubated HAECs with STO-609 before exposing them to liraglutide. STO-609 is a CaMKKα and CaMKKβ specific inhibitor that has been used in other studies to inhibit the phosphorylation of AMPK resulting from calcium fluxes [39]. Pre-incubation with STO-609 effectively inhibited the phosphorylation of CaMK1 and AMPK (Figure 5A, 5B) and their downstream targets pCREB and peNOS (Figure S2). In addition, STO-609 blocked the effects of liraglutide on TNFα and LPS induced monocyte adhesion (Figure 5E, 5F) demonstrating that this anti-inflammatory benefit is also dependent on activation of CaMKKβ.

### ShRNA Mediated Knockdown of AMPK Prevents the Anti-inflammatory Effect of Liraglutide

**Figure 6:**
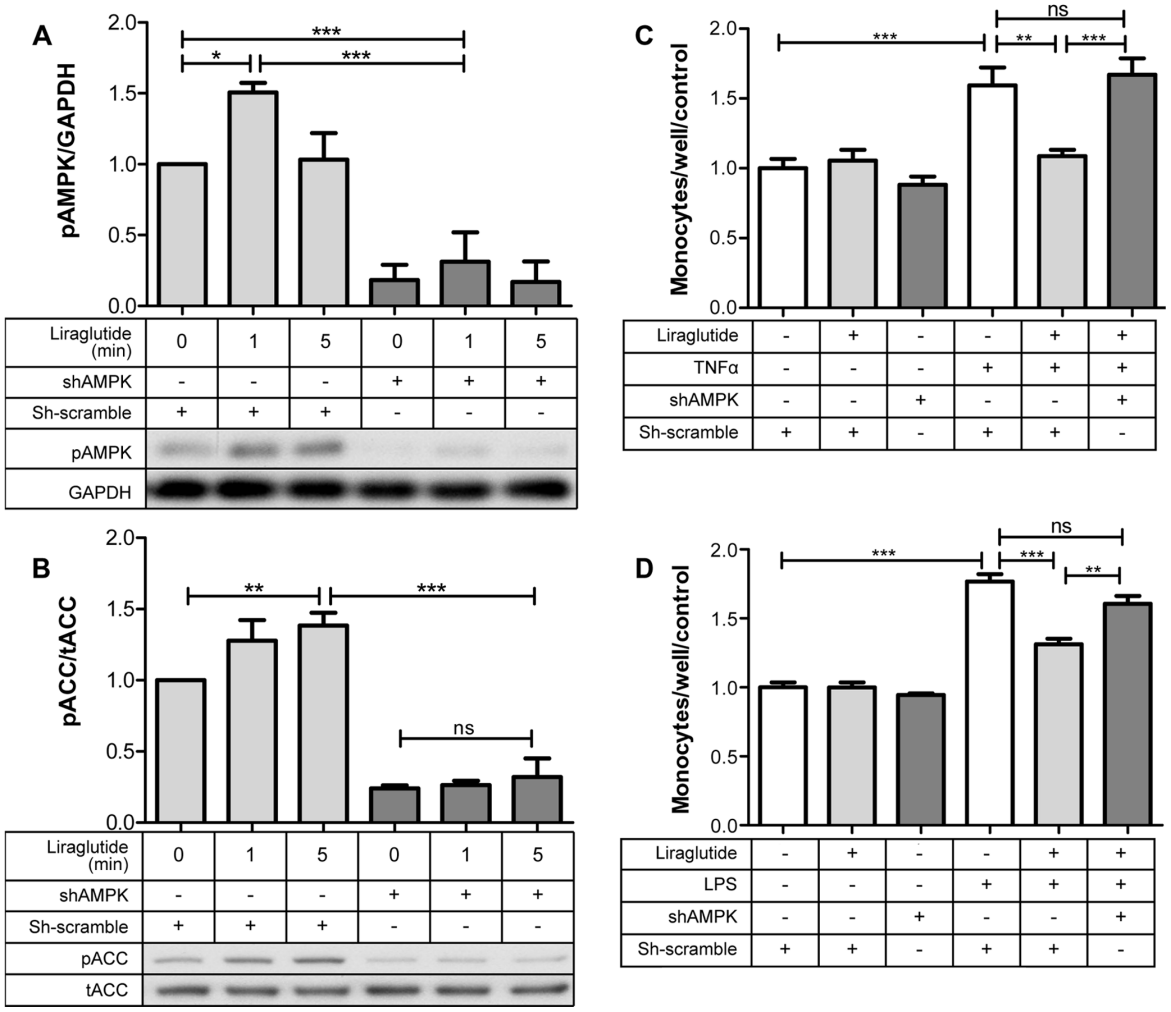
shAMPK knocks down AMPK and inhibits the effects of liraglutide on AMPK and ACC phosphorylation, and monocyte adhesion. **A:** AMPK phosphorylation (Thr172) **B:** ACC phosphorylation (Ser79). **C,D:** Infection of HAECs with shAMPK inhibited the liraglutide induced decrease in TNFα (10 ng/mL) and LPS (2 µg/mL ) stimulated monocyte adhesion (n = 6). *p<0.05, **p<0.01, ***p<0.001.

With the use of a lentivirus to express a shAMPK, we knocked down the expression of AMPK in HAECs. The latter was evident both microscopically, as the virus co-expressed a GFP tag allowing for visualization of infection (Figure S3), and by significantly reduced (83%) AMPK protein levels (Figure S3). Additionally, the lentivirus had no effect on the cellular density, as indicated by total cellular protein quantification (Figure S3). The expression of shAMPK prevented liraglutide induced activation of AMPK (Figure 6A) and its downstream target pACC (Figure 6B) as well as the anti-inflammatory effects of liraglutide on monocyte adhesion (Figure 6C, 6D). These results indicate both that liraglutide activates AMPK, and that the latter is key to inhibiting monocyte adhesion, a putative early step in atherogenesis.

## Discussion

**Figure 7:**
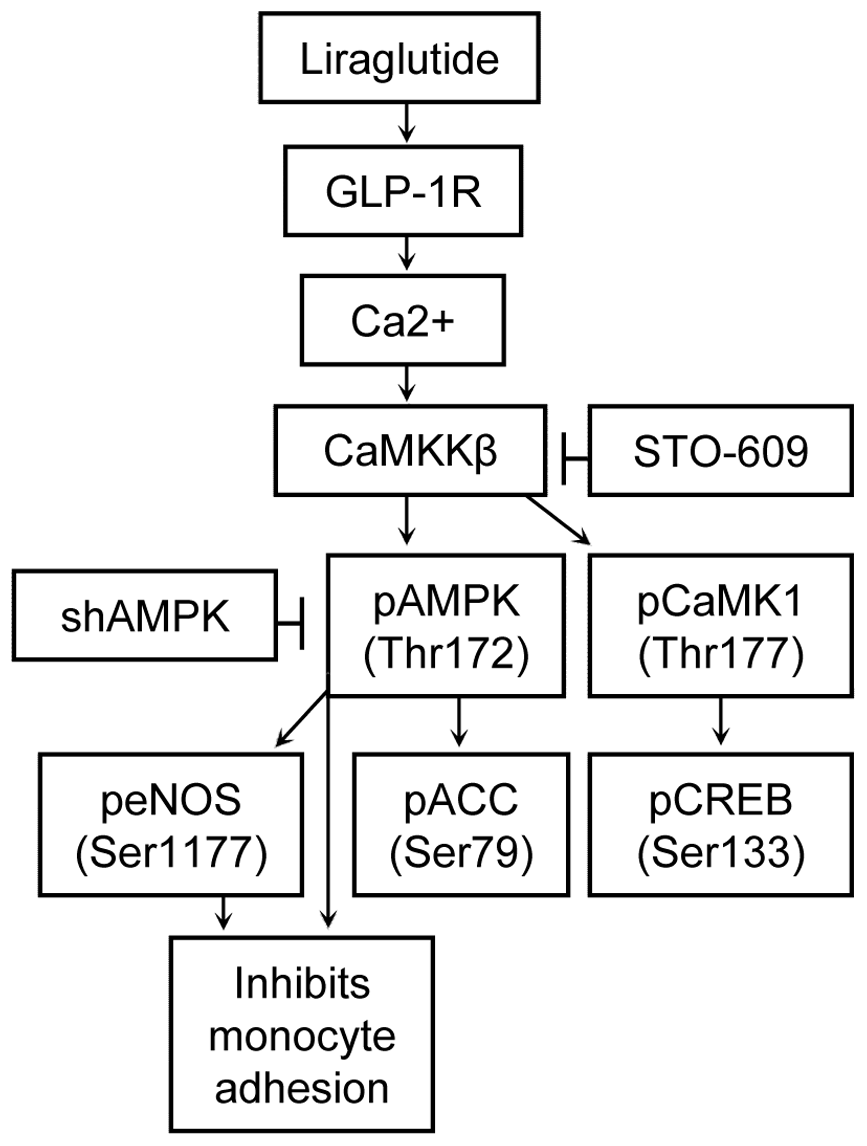
Schema depicting the proposed mechanisms for the anti-inflammatory effect of liraglutide on human aortic endothelial cells.

It has been suggested by epidemiological [11], [12], [13] and clinical studies [14], [16], [24], [40] that treatment with a GLP-1 receptor agonist, such as liraglutide, diminishes the risk of cardiovascular disease in patients with Type 2 diabetes. The mechanism, however, by which it confers this benefit, is incompletely understood. The results of the present study suggest that liraglutide may act by inhibiting chronic inflammation in human aortic endothelial cells, an initiating event in atherogenesis. Thus we found that liraglutide diminishes both TNFα and LPS induced E-selectin and VCAM-1 expression, and the increase in monocyte adhesion that accompanies it. The results also demonstrate that liraglutide produces these effects by increasing cell Ca^2+^ and secondarily the activities of CAMKKβ and AMPK (Figure 7).

As recently reviewed, TNFα and LPS stimulated monocyte adhesion to endothelial cells are initiating events in the pathogenesis of atherosclerosis [41]. Therefore, our findings that liraglutide reduces the increased expression of VCAM-1 and E-selectin, and monocyte adhesion, caused by TNFα and LPS in HAECs (Figure 1A–D) could be highly relevant. Others have found that liraglutide inhibits high-glucose induced endoplasmic reticulum stress [42], and also TNFα induced markers of endothelial dysfunction such as PAI-1 [43], ICAM-1, and VCAM-1 in human umbilical vein endothelial cells (HUVECs) [44]. However, whether these effects could functionally affect endothelial cell monocyte interaction was not known. This is the first study demonstrating that liraglutide directly diminishes monocyte adhesion to inflamed primary human aortic endothelial cells and find the mechanism by which this occurs. In healthy vasculature, diapedesis is important for immune protection against infection. In contrast, under atherogenic conditions, monocytes diapedese, engulf excessive lipids, become foam cells and adhere to the intimal space. This generates a feed forward cycle of inflammation, increasing adhesion and diapedesis of monocytes, and formation of an atheroma [45]. If, as shown here, liraglutide downregulates expression of adhesion molecules in endothelial cells, and subsequent to this, monocyte adhesion, it presumably is inhibiting atherogenesis.

As already noted, the mechanism through which GLP-1R agonists, such as liraglutide, affect vascular endothelium has not been well studied. In pancreatic beta cells, they primarily signal through cAMP and calcium dependent mechanisms [30], [31]. We demonstrate here that stimulating HAECs with liraglutide (100 nM) results in a significant increase in intracellular calcium (Figure 3). To our knowledge, this is the first demonstration that liraglutide causes an increase in intracellular calcium in any cell type. The effects of liraglutide on intracellular calcium release were also shown to be dose dependent as high dose liraglutide (2500 nM) treatment increased intracellular calcium to a greater extent (Figure 3D). Although liraglutide also increased cAMP levels, its effect at the concentration found in the circulation of patients (100 nM) was small (Figure S1). Intriguingly, in beta cells isolated from Wistar rats, GLP-1 treatment was shown to rapidly increase intracellular calcium levels [46] similar to the effect we observed with liraglutide. Whether this effect of GLP-1 and liraglutide on calcium is dependent on stimulation of the known GLP-1 receptor is unclear and a potential line of future investigation. However, liraglutide’s inhibition of TNFα induced expression of ICAM-1 and VCAM-1 has been shown to be GLP-1R dependent [47].

The anti-inflammatory effects of liraglutide on endothelial cells observed in this study are similar to those observed previously by our lab following AMPK activation in HUVECs [48]. In the latter, E-selectin and VCAM1 were not measured; however, AMPK inhibited TNFα induced NFκB activation, and decreased the expression of cellular adhesion molecules [48]. This is a key observation as cellular adhesion molecules generally require NFκB for transcription. In this study we found that liraglutide activated AMPK and its substrate eNOS (Figure 4A, 4C). Activation of eNOS by AMPK increased nitric oxide (NO) synthesis [49], which scavenges superoxides [50], induces vasodilation [51] and inhibits leukocyte adhesion [52]. Thus, the observed activation of AMPK by liraglutide (Figure 4A) suggests a potential mechanism for the anti-inflammatory effects previously discussed.

There are two primary mechanisms for the activation of AMPK in endothelial cells, one through CaMKKβ [53] and the other LKB1 [54]. CaMKKβ is activated by an influx of calcium into the cytosol, an effect we have observed with liraglutide treatment, and which can occur independently of LKB1 [55]. In addition to activating AMPK, CaMKKβ phosphorylates and activates CaMKI at Thr 177 leading to its activation. Thus, it is noteworthy that liraglutide increased the phosphorylation of CREB at Ser 133, a known target of CaMKI [37] (Figure 4E, see also Figure 7). Although it is possible that the small observed increase in cAMP led to the activation of PKA, which also phosphorylates CREB at Ser 133, inhibition of CaMKKβ blocked the phosphorylation of CREB (Figure S3) suggesting that CaMK1 activity was most likely responsible for this effect.

We discovered that activation of CaMKKβ by liraglutide is necessary for phosphorylation of AMPK and CaMK1 through use of STO-609, a CaMKKα and CaMKKβ specific inhibitor. STO-609 effectively blocked liraglutide stimulated phosphorylation of AMPK and CaMK1 (Figure 5A,B) and their targets, peNOS and pCREB (Figure S3). Additionally, STO-609 blocked the anti-inflammatory effects of liraglutide in that it prevented TNFα and LPS induced monocyte adhesion (Figure 5E, 5F). From this data we can conclude that liraglutide signals through CaMKKβ to activate CaMK1 and AMPK (Figure 7), and that this confers protection against TNFα and LPS induced monocyte adhesion.

To determine whether or not the anti-inflammatory effects of liraglutide are also dependent on activation of AMPK, we knocked down AMPK expression with a lentivirus shAMPK. ShAMPK expression inhibited the ability of liraglutide to induce phosphorylation of AMPK at T172 (Figure 6A) and of the AMPK target ACC at S79 (Figure 6B). Importantly, it also prevented the ability of liraglutide to inhibit TNFα and LPS stimulated monocyte adhesion (Figure 6C, 6D). Therefore, the anti-inflammatory effects of liraglutide on human aortic endothelial cells are dependent on the activation of AMPK, as shown in Figure 7.

The results of the present study suggest that activation of CaMKKβ and AMPK by liraglutide could explain its reported cardiovascular benefits in patients with Type 2 diabetes. Interestingly, cardiovascular benefits of GLP-1 mimetics have also been found independent of diabetes. GLP-1 mimetic therapy improved global left ventricular (LV) function in patients with acute myocardial infarction and LV dysfunction after successful reperfusions [56], and ischemia in patients with coronary artery disease [57]. Unlike many other diabetes treatments, liraglutide does not create an added risk of hypoglycemia. Thus, it also could prove useful in non-diabetic patients at risk for atherosclerotic cardiovascular disease.

## Supporting Information

Figure S1 **Effect of 3 minute incubation with 100nM or 1000** **nM liraglutide on cAMP levels in HAECs (n = 10).** *p<0.05, ***p<0.001.
(TIF)

Figure S2 **STO-609 inhibits the phosphorylation of targets of AMPK (eNOS), and CaMK1 (CREB).** **A:** peNOS (Ser177) **B:** pCREB (Ser133). Liraglutide (100 nM), STO-609 (0.5 µg/mL). *p<0.05, **p<0.01, ***p<0.001.
(TIF)

Figure S3 **shAMPK knocks down AMPK expression.** **A,B:** 20× Micrographs of HAECs under bright field (A) and fluorescence to show the presence of GFP indicating positive virus infection (B). **C:** Quantification of western blot of total AMPK protein level normalized to GAPDH shows 82% reduction in tAMPK (n = 6)**.** **D:** Quantification of total cellular protein levels indicating virus does not affect cell density (n = 6). *p<0.05, **p<0.01, ***p<0.001.
(TIF)

## References

[1] Drucker D, Philippe J, Mojsov S, Chick W, Habener J (1987) Glucagon- like peptide 1 stimulates insulin gene expression and increases cyclic AMP levels in a rat islet cell line. Proc Natl Acad Sci USA 84: 3434–3438. 3033647 10.1073/pnas.84.10.3434 PMC304885

[2] Kreymann B, Ghatei M, Williams G, Bloom S (1987) Glucagon-like peptide-1 7–36: A physiological incretin in man. Lancet 2: 1300–1304. 2890903 10.1016/s0140-6736(87)91194-9

[3] Russell-Jones D (2009) Molecular, pharmacological and clinical aspects of liraglutide, a once-daily human GLP-1 analogue. Mol Cell Endocrinol 297: 137–140. 19041364 10.1016/j.mce.2008.11.018

[4] Nauck M, El-Ouaghlid A, Hompesch M, Jacobsen J, Elbrond B (2003) No impairment of hypoglycemia counterregulation via glucagon with NN2211, a GLP-1 derivative, in subjects with type 2-diabetes. Diabetes 52: A128.

[5] Niswender K, Pi-Sunyer X, Buse J, Jensen K, Toft A, et al (2013) Weight change with liraglutide and comparator therapies: an analysis of seven phase 3 trials from the liraglutide diabetes development programme. Diabetes Obes Metab 15: 42–54. 22862847 10.1111/j.1463-1326.2012.01673.x

[6] Flint A, Kapitza C, Hindsberger C, Zdravkovic M (2011) The once-daily human glucagon-like peptide-1 (GLP-1) analog liraglutide improves postprandial glucose levels in type 2 diabetes patients. Advances in Therapy 28: 213–226. 21340616 10.1007/s12325-010-0110-x

[7] Horowitz M, Flint A, Jones K, Hindsberger C, Rasmussen M, et al (2012) Effect of the once-daily human GLP-1 analogue liraglutide on appetite, energy intake, energy expenditure and gastric emptying in type 2 diabetes. Diabetes Res Clin Pract 97: 258–266. 22446097 10.1016/j.diabres.2012.02.016

[8] van Can J, Sloth B, Jensen C, Flint A, Blaak E, et al.. (2013) Effects of the once-daily GLP-1 analog liraglutide on gastric emptying, glycemic parameters, appetite and energy metabolism in obese, non-diabetic adults. Int J Obese (Lond). 10.1038/ijo.2013.162 PMC4052428 23999198

[9] Cuthbertson D, Irwin A, Gardner C, Daousi C, Purewal T, et al (2012) Improved glycaemia correlates with liver fat reduction in obese, type 2 diabetes, patients given glucagon-like peptide-1 (GLP-1) receptor agonists. PLos One 7: e50117. 23236362 10.1371/journal.pone.0050117 PMC3516516

[10] Forst T, Michelson G, Ratter F, Weber M, Anders S, et al (2012) Addition of liraglutide in patients with Type 2 diabetes well controlled on metformin monotherapy improves several markers of vascular function. Diabet Med 29: 1115–1118. 22288732 10.1111/j.1464-5491.2012.03589.x

[11] Best J, Hoogwerf B, Herman W, Pelletier E, Smith D, et al (2011) Risk of cardiovascular disease events in patients with type 2 diabetes prescribed the GLP-1 receptor agonist exenatide twice daily or other glucose-lowering therapies: a retrospective analysis of the LifeLink database. Diabetes Care 34: 90–95. 20929995 10.2337/dc10-1393 PMC3005487

[12] Horton E, Silberman C, Davis K, Berria R (2010) Weight Loss, Glycemic Control, and Changes in Cardiovascular Biomarkers in Patients With Type 2 Diabetes Receiving Incretin Therapies or Insulin in a Large Cohort Database. Diabetes Care 33: 1759–1765. 20460445 10.2337/dc09-2062 PMC2909058

[13] Marso S, Lindsey J, Stolker J, House J, Martinez Ravn G, et al (2011) Cardiovascular safety of liraglutide assessed in a patient-level pooled analysis of phase 2: 3 liraglutide clinical development studies. Diab Vasc Dis Res 8: 237–240. 21653676 10.1177/1479164111408937

[14] Moretto T, Milton D, Ridge T, Macconell L, Okerson T, et al (2008) Efficacy and Tolerability of Exenatide Monotherapy Over 24 Weeks in Antidiabetic Drug-Naïve Patients with Type 2 Diabetes: A Randomized, Double-Blind, Placebo-Controlled Parallel-Group Study. Clin Ther 30: 1448–1460. 18803987 10.1016/j.clinthera.2008.08.006

[15] Ceriello A, Esposito K, Testa R, Bonfigli A, Marra M, et al (2011) The possible protective role of glucagon-like peptide 1 on endothelium during the meal and evidence for an “endothelial resistance” to glucagon-like peptide 1 in diabetes. Diabetes Care 34: 697–702. 21273492 10.2337/dc10-1949 PMC3041210

[16] Koska J, Schwartz E, Mullin M, Schwenke D, Reaven P (2010) Improvement of postprandial endothelial function after a single dose of exenatide in individuals with impaired glucose tolerance and recent-onset type 2 diabetes. Diabetes Care 33: 1028–1030. 20200309 10.2337/dc09-1961 PMC2858168

[17] Creely S, McTernan P, Kusminski C, Fisher F, Da Silva N, et al (2007) Lipopolysaccharide activates an innate immune system response in human adipose tissue in obesity and type 2 diabetes. Am J Physiol Endocrinol Metab 292: E740–747. 17090751 10.1152/ajpendo.00302.2006

[18] Kelly C, Colgan S, Frank D (2012) Of microbes and meals: the health consequences of dietary endotoxemia. Nutr Clin Pract 27: 215–225. 22378797 10.1177/0884533611434934 PMC4046172

[19] Pussinen P, Tuomisto K, Jousilahti P, Havulinna A, Sundvall J, et al (2007) Endotoxemia, immune response to periodontal pathogens, and systemic inflammation associate with incident cardiovascular disease events. Arterioscler Thromb Vasc Biol 27: 1433–1439. 17363692 10.1161/ATVBAHA.106.138743

[20] Chen C, Khismatullin D (2013) Synergistic effect of histamine and TNF-α on monocyte adhesion to vascular endothelial cells. Inflammation 36: 309–319. 23053728 10.1007/s10753-012-9548-0

[21] Lee W, Yang E, Ku S, Song K, Bae J (2013) Anti-inflammatory effects of oleanolic acid on LPS-induced inflammation in vitro and in vivo. Inflammation 36: 94–102. 22875543 10.1007/s10753-012-9523-9

[22] Westhorpe C, Dufour E, Maisa A, Jaworowski A, Crowe S, et al (2012) Endothelial cell activation promotes foam cell formation by monocytes following transendothelial migration in an in vitro model. Exp Mol Pathol 93: 220–226. 22609311 10.1016/j.yexmp.2012.03.014 PMC3408785

[23] Bullock B, Heller R, Habener J (1996) Tissue distribution of messenger ribonucleic acid encoding the rat glucagon-like peptide-1 receptor. Endocrinology 137: 2968–2978. 8770921 10.1210/endo.137.7.8770921

[24] Nyström T, Gutniak M, Zhang Q, Zhang F, Holst J, et al (2004) Effects of glucagon-like peptide-1 on endothelial function in type 2 diabetes patients with stable coronary artery disease. American Journal of Physiology and Endocrinology 287: E1209–E1215. 10.1152/ajpendo.00237.2004 15353407

[25] Drucker D (2006) The biology of incretin hormones. Cell Metabolism 3: 153–165. 16517403 10.1016/j.cmet.2006.01.004

[26] Nordisk N (2014) PRODUCT MONOGRAPH: Victoza/Liraglutide. Mississauga, Ontario. Available: http://novonordiskca/PDF_Files/our_products/Victoza/Victoza_PM_ENpdf.

[27] Woollard K (2013) Immunological aspects of atherosclerosis. Clin Sci (Lond) 125: 221–235. 23668229 10.1042/CS20120576

[28] Galkina E, Ley K (2007) Vascular adhesion molecules in atherosclerosis. Arterioscler Thromb Vasc Biol 27: 2292–2301. 17673705 10.1161/ATVBAHA.107.149179

[29] Qin Z (2012) The use of THP-1 cells as a model for mimicking the function and regulation of monocytes and macrophages in the vasculature. Atherosclerosis 221: 2–11. 21978918 10.1016/j.atherosclerosis.2011.09.003

[30] Cullinan C, Brady E, Saperstein R, Leibowitz M (1994) Glucose-dependent alterations of intracellular free calcium by glucagon-like peptide-1(7–36 amide) in individual ob/ob mouse beta-cells. Cell Calcium 15: 391–400. 8033197 10.1016/0143-4160(94)90014-0

[31] Holz G (2004) Epac: A new cAMP-binding protein in support of glucagon-like peptide-1 receptor-mediated signal transduction in the pancreatic beta-cell. Diabetes 53: 5–13. 14693691 10.2337/diabetes.53.1.5 PMC3012130

[32] Kwan H, Huang Y, Yao X, Leung F (2009) Role of cyclic nucleotides in the control of cytosolic Ca2+ levels in vascular endothelial cells. Clin Exp Pharmacol Physiol 36: 857–866. 19413591 10.1111/j.1440-1681.2009.05199.x

[33] Tran Q, Ohashi K, Watanabe H (2000) Calcium signalling in endothelial cells. Cardiovascular Research 48: 13–22. 11033104 10.1016/s0008-6363(00)00172-3

[34] Yamamizu K, Yamashita J (2011) Roles of cyclic adenosine monophosphate signaling in endothelial cell differentiation and arterial-venous specification during vascular development. Circ J 75: 253–260. 21178292 10.1253/circj.cj-10-0915

[35] Xing J, Wang Q, Coughlan K, Viollet B, Moriasi C, et al (2013) Inhibition of AMP-activated protein kinase accentuates lipopolysaccharide-induced lung endothelial barrier dysfunction and lung injury in vivo. American Journal of Pathology 182: 1021–1030. 23306156 10.1016/j.ajpath.2012.11.022 PMC3589075

[36] Hawley S, Selbert M, Goldstein E, Edelman A, Carling D, et al (1995) 5'-AMP activates the AMP-activated protein kinase cascade, and Ca2+/calmodulin activates the calmodulin-dependent protein kinase I cascade, via three independent mechanisms. J Biol Chem 270: 27186–27191. 7592975 10.1074/jbc.270.45.27186

[37] Francis H, Glaser S, Demorrow S, Gaudio E, Ueno Y, et al (2008) Small mouse cholangiocytes proliferate in response to H1 histamine receptor stimulation by activation of the IP3/CaMK I/CREB pathway. Am J Physiol Cell Physiol 295: C499–513. 18508907 10.1152/ajpcell.00369.2007 PMC2518416

[38] Nakamura K, Kamouchi M, Arimura K, Nishimura A, Kuroda J, et al (2012) Extracellular acidification activates cAMP responsive element binding protein via Na+/H+ exchanger isoform 1-mediated Ca^2+^ oscillation in central nervous system pericytes. Arterioscler Thromb Vasc Biol 32: 2670–2677. 22922957 10.1161/ATVBAHA.112.254946

[39] Woods A, Dickerson K, Heath R, Hong S, Momcilovic M, et al (2005) Ca2+/calmodulin-dependent protein kinase kinase-β acts upstream of AMP-activated protein kinase in mammalian cells. Cell Metabolism 2: 21–33. 16054096 10.1016/j.cmet.2005.06.005

[40] Sokos G, Nikolaidis L, Mankad S, Elahi D, Shannon R (2006) Glucagon-Like Peptide-1 Infusion Improves Left Ventricular Ejection Fraction and Functional Status in Patients with Chronic Heart Failure. J Card Fail 12: 694–699. 17174230 10.1016/j.cardfail.2006.08.211

[41] Libby P, Ridker P, Hansson G (2011) Progress and challenges in translating the biology of atherosclerosis. Nature 473: 317–325. 21593864 10.1038/nature10146

[42] Schisano B, Harte A, Lois K, Saravanan P, Al-Daghri N, et al (2012) GLP-1 analogue, Liraglutide protects human umbilical vein endothelial cells against high glucose induced endoplasmic reticulum stress. Regulatory Peptides 10: 46–52. 10.1016/j.regpep.2011.11.008 22120833

[43] Liu H, Hu Y, Simpson R, Dear A (2008) Glucagon-like peptide-1 attenuates tumour necrosis factor-alpha-mediated induction of plasminogen [corrected] activator inhibitor-1 expression. J Endocrinol 196: 57–65. 18180317 10.1677/JOE-07-0387

[44] Shiraki A, Oyama J, Komoda H, Asaka M, Komatsu A, et al (2012) The glucagon-like peptide 1 analog liraglutide reduces TNF-a-induced oxidative stress and inflammation in endothelial cells. Atherosclerosis 221: 375–382. 22284365 10.1016/j.atherosclerosis.2011.12.039

[45] Tuttolomondo A, Di Raimondo D, Pecoraro R, Arnao V, Pinto A, et al (2012) Atherosclerosis as an inflammatory disease. Curr Pharm Des 18: 4266–4288. 22390643 10.2174/138161212802481237

[46] Damdindorj B, Dezaki K, Kurashina T, Sone H, Rita R, et al (2012) Exogenous and endogenous ghrelin counteracts GLP-1 action to stimulate cAMP signaling and insulin secretion in islet β-cells. FEBS Lett 586: 2555–2562. 22750144 10.1016/j.febslet.2012.06.034

[47] Gaspari T, Liu H, Welungoda I, Hu Y, Widdop R, et al (2011) A GLP-1 receptor agonist liraglutide inhibits endothelial cell dysfunction and vascular adhesion molecule expression in an ApoE−/− mouse model. Diabetes and Vascular Disease Research 8: 117–124. 21562063 10.1177/1479164111404257

[48] Cacicedo J, Yagihashi N, Keaney JJ, Ruderman N, Ido Y (2004) AMPK inhibits fatty acid-induced increases in NF-kB transactivation in cultured human umbilical vein endothelial cells. Biochemical and Biophysical Research Communications 324: 1204–1209. 15504342 10.1016/j.bbrc.2004.09.177

[49] Morrow V, Foufelle F, Connell J, Petrie J, Gould G, et al (2003) Direct activation of AMP-activated protein kinase stimulates nitric-oxide synthesis in human aortic endothelial cells. J Biol Chem 278: 31629–31639. 12791703 10.1074/jbc.M212831200

[50] Aoki C, Suzuki K, Yanagi K, Satoh H, Niitani M, et al (2012) Miglitol, an anti-diabetic drug, inhibits oxidative stress-induced apoptosis and mitochondrial ROS over-production in endothelial cells by enhancement of AMP-activated protein kinase. J Pharmacol Sci 120: 121–128. 23018899 10.1254/jphs.12108fp

[51] Wu Y, Zhang C, Dong Y, Wang S, Song P, et al (2012) Activation of the AMP-activated protein kinase by eicosapentaenoic acid (EPA, 20: 5 n−3) improves endothelial function in vivo. PLOS One 7: e35508. 22532857 10.1371/journal.pone.0035508 PMC3330125

[52] Ou H, Lee W, Wu C, Chen J, Sheu W (2012) Aspirin prevents resistin-induced endothelial dysfunction by modulating AMPK, ROS, and Akt/eNOS signaling. J Vasc Surg 55: 1104–1115. 22244860 10.1016/j.jvs.2011.10.011

[53] Stahmann N, Woods A, Carling D, Heller R (2006) Thrombin Activates AMP-Activated Protein Kinase in Endothelial Cells via a Pathway Involving Ca2+/Calmodulin-Dependent Protein Kinase Kinase β. Mol Cell Biol 26: 5933–5945. 16880506 10.1128/MCB.00383-06 PMC1592798

[54] Hawley S, Boudeau J, Reid J, Mustard K, Udd L, et al (2003) Complexes between the LKB1 tumor suppressor, STRAD alpha/beta and MO25 alpha/beta are upstream kinases in the AMP-activated protein kinase cascade. J Biol 2: 28. 14511394 10.1186/1475-4924-2-28 PMC333410

[55] Hurley R, Anderson K, Franzone J, Kemp B, Means A, et al (2005) The Ca2+/calmodulin-dependent protein kinase kinases are AMP-activated protein kinase kinases. J Biol Chem 280: 29060–29066. 15980064 10.1074/jbc.M503824200

[56] Nikolaidis L, Mankad S, Sokos G, Miske G, Shah A, et al (2004) Effects of Glucagon-Like Peptide-1 in Patients with Acute Myocardial Infarction and Left Ventricular Dysfunction After Successful Reperfusion. Circulation 109: 962–965. 14981009 10.1161/01.CIR.0000120505.91348.58

[57] Read P, Khan F, Dutka D (2012) Cardioprotection against ischaemia induced by dobutamine stress using glucagon-like peptide-1 in patients with coronary artery disease. Heart 98: 408–413. 21561896 10.1136/hrt.2010.219345

[58] Krasner N, Ido Y, Ruderman N, Cacicedo J (2011) Glucagon-like peptide-1 (GLP-1) activates AMPK and diminishes inflammation in human aortic endothelial cells; Orlando, FL.

