# Supplementary figures and images for: Glucagon-Like Peptide-1 (GLP-1) Analog Liraglutide Inhibits Endothelial Cell Inflammation through a Calcium and AMPK Dependent Mechanism
Source: PLoS One. 2014 May 16;9(5):e97554. doi: 10.1371/journal.pone.0097554 (PMC4023984; doi:10.1371/journal.pone.0097554)

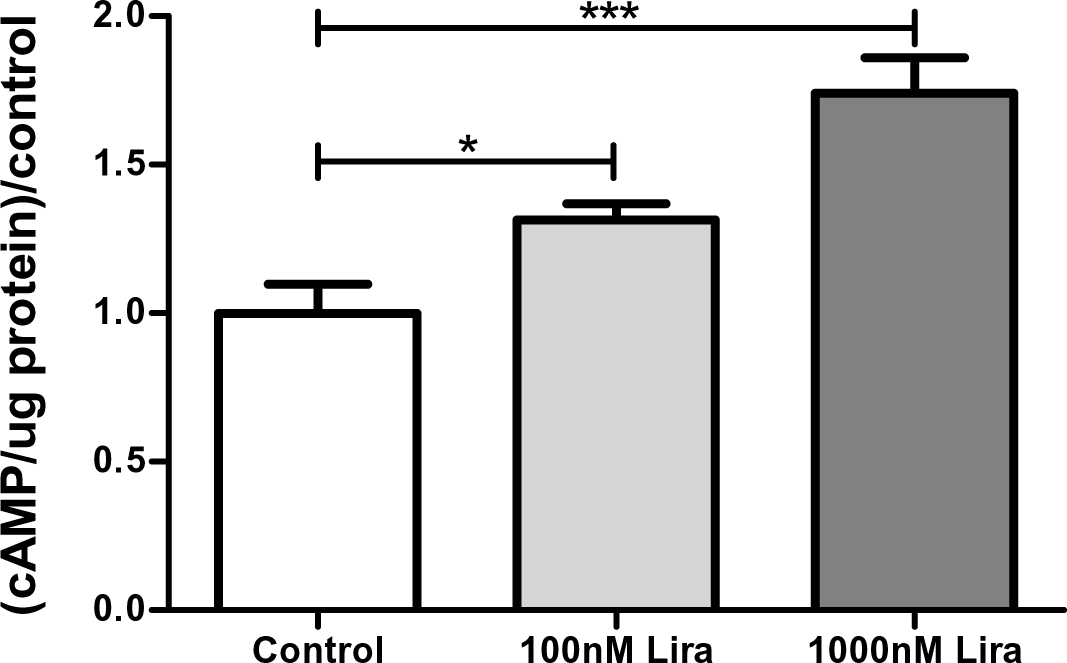

Supplement: Figure S1 — Effect of 3 minute incubation with 100nM or 1000 nM liraglutide on cAMP levels in HAECs (n = 10). *p<0.05, ***p<0.001. (TIF) [file pone.0097554.s001.tif]

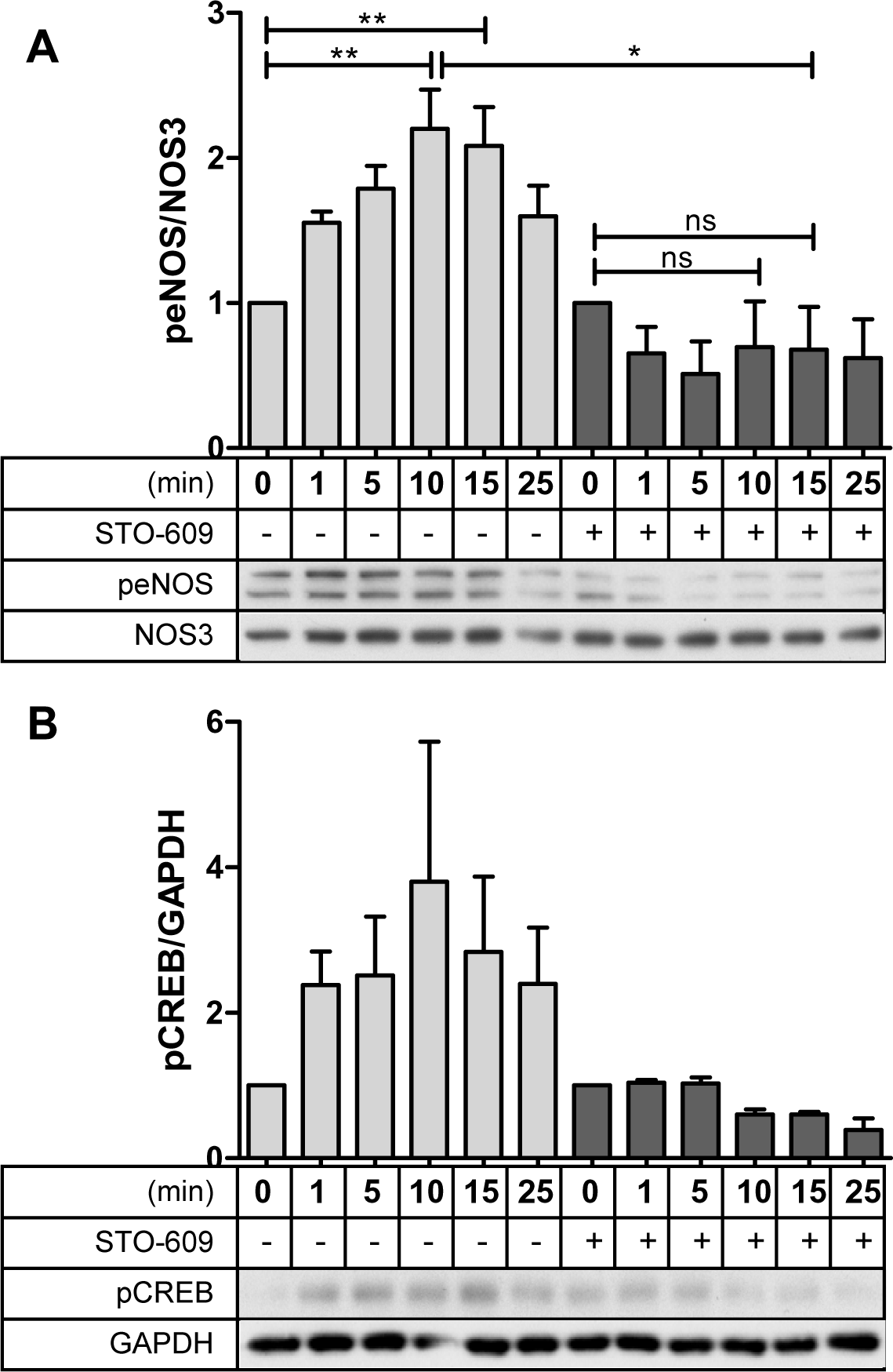

Supplement: Figure S2 — STO-609 inhibits the phosphorylation of targets of AMPK (eNOS), and CaMK1 (CREB). A: peNOS (Ser177) B: pCREB (Ser133). Liraglutide (100 nM), STO-609 (0.5 µg/mL). *p<0.05, **p<0.01, ***p<0.001. (TIF) [file pone.0097554.s002.tif]

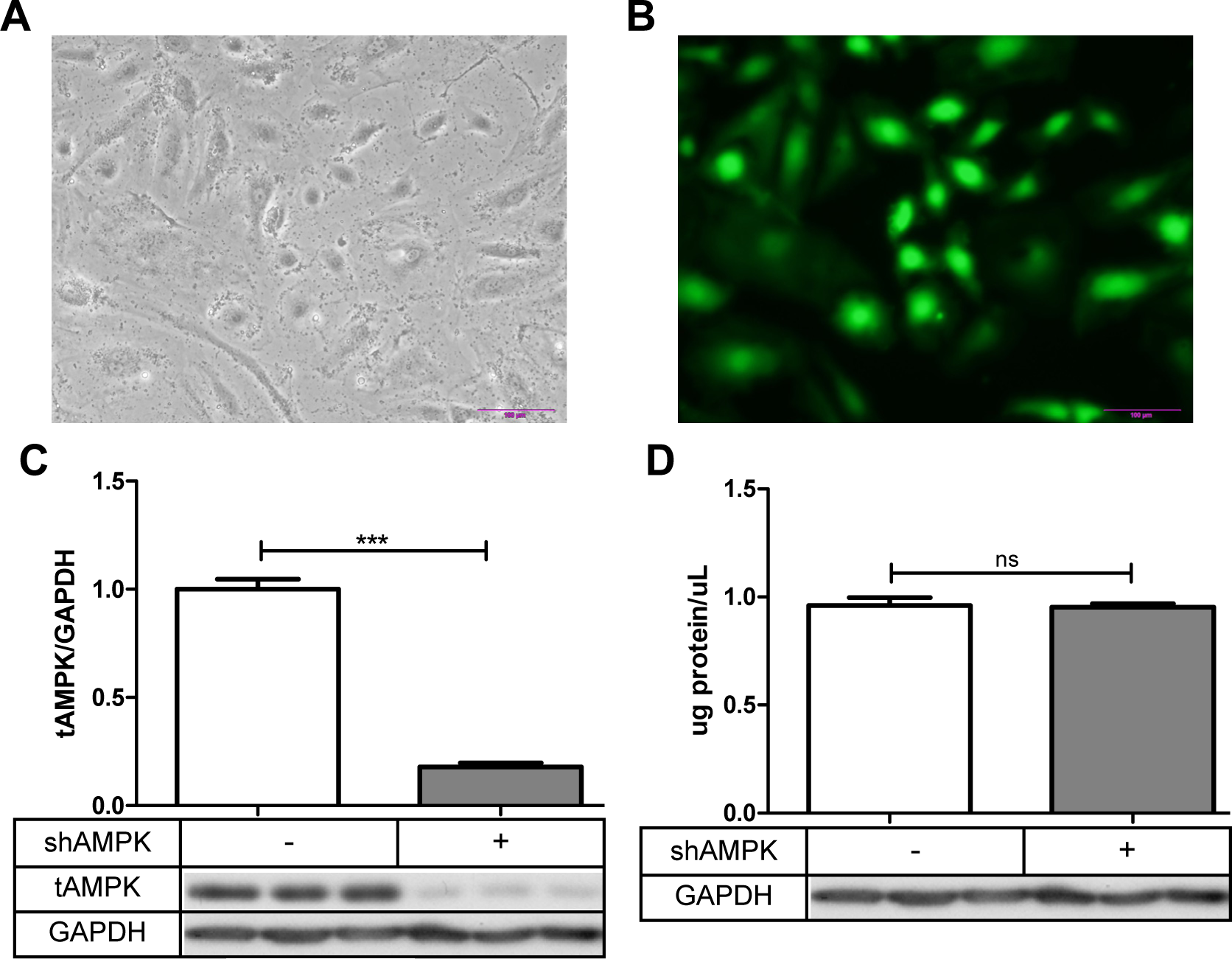

Supplement: Figure S3 — shAMPK knocks down AMPK expression. A,B: 20× Micrographs of HAECs under bright field (A) and fluorescence to show the presence of GFP indicating positive virus infection (B). C: Quantification of western blot of total AMPK protein level normalized to GAPDH shows 82% reduction in tAMPK (n = 6). D: Quantification of total cellular protein levels indicating virus does not affect cell density (n = 6). *p<0.05, **p<0.01, ***p<0.001. (TIF) [file pone.0097554.s003.tif]
